# Advancing Evidence-Based Practice Through Social Movement Strategies: A Case Study in Healthcare Transformation

**DOI:** 10.3390/healthcare14101358

**Published:** 2026-05-15

**Authors:** Evalyn Abalos, Theresa Guino-o, Freslyn Lim-Saco, May Ross Café, Theorose June Bustillo, Kathleah Caluscusan, Maria Theresa Belciña, Veveca Bustamante, Rozzano Locsin

**Affiliations:** 1 College of Nursing, Silliman University, Dumaguete City 6200, Philippines; evalyneabalos@su.edu.ph (E.A.); theresaaguino-o@su.edu.ph (T.G.-o.); freslynslim@su.edu.ph (F.L.-S.); maypcafe@su.edu.ph (M.R.C.); theoroseqbustillo@su.edu.ph (T.J.B.); kathleahscaluscusan@su.edu.ph (K.C.); vevecavbustamante@su.edu.ph (V.B.); 2 Christine E. Lynn College of Nursing, Florida Atlantic University, 777 Glades Rd., Boca Raton, FL 33431, USA

**Keywords:** evidence-based practice implementation, social movement strategies, implementation science, clinical guideline implementation, knowledge translation, low- and middle-income countries

## Abstract

**Highlights:**

- Social movement strategies strengthen evidence-based practice implementation.
- Collective ownership and distributed leadership support sustained change.
- Multilevel engagement enhances organizational readiness for implementation.
- Socially driven approaches enable scalable healthcare practice improvement.
- Findings provide actionable guidance for strengthening health systems.

**Abstract:**

Background: The importance of evidence-based practice (EBP) is well recognized, yet its implementation remains challenging across healthcare systems, especially in low- and middle-income countries, where resource constraints, workforce turnover, and organizational barriers can hinder practice change. The traditional approach to implementation has focused on training, guidelines, and leadership support; however, these strategies do not always sustain frontline staff engagement. Objective: This descriptive case study examined how social movement strategies supported a multi-year EBP implementation initiative within a Philippine academic–clinical partnership. Methods: Program documents, training records, implementation reports, curriculum materials, and internal records of guideline-related activities were reviewed. Data were organized using the Social Movement Action Framework, with attention to preconditions for change, social movement mechanisms, and implementation outcomes. Results: The initiative included champion training, guideline integration, awareness activities, academic–clinical collaboration, and practice-focused implementation efforts related to breastfeeding, vascular access device management, and pressure injury prevention. These activities provided observable process indicators of stakeholder engagement, shared ownership, and continued use of guideline-informed practices. Conclusions: Social movement strategies may offer a useful complementary lens for understanding how EBP implementation gains momentum in real-world healthcare settings. Additional studies should explore their relationship to implementation outcomes and clinical care processes.

## 1. Introduction

The integration of evidence-based practice (EBP) into the routine of health care is critical to optimizing patient outcomes and quality of care, and to minimizing unwarranted variation in clinical practice. Health systems continue to struggle to translate research into everyday practice despite the abundance of clinical guidelines and implementation frameworks. Implementation science has helped address this gap by identifying strategies that support the adoption, delivery, and sustainability of evidence-based interventions in real-world settings [1,2].

These challenges can be more pronounced in low- and middle-income countries, where implementation may be affected by limited resources, workforce turnover, competing institutional priorities, and differences in local context [3]. In these settings, implementation requires more than disseminating guidelines or delivering training. It also requires attention to leadership, readiness for change, stakeholder engagement, and the social conditions that allow practice change to take hold [4,5].

Many established implementation frameworks, including the Consolidated Framework for Implementation Research, the Promoting Action on Research Implementation in Health Services framework, and the Medical Research Council framework for complex interventions, help explain how context, evidence, facilitation, and intervention complexity influence implementation [6,7,8]. These frameworks allow the identification of barriers, facilitators, and implementation conditions. However, these frameworks may give less attention to how implementation efforts build momentum over time through collective will, common identity, peer influence, and grassroots leadership. The process can also be seen as a social movement.

Social movement theory offers a complementary way to understand this process. Rather than viewing implementation only as a technical or organizational task, a social movement perspective emphasizes shared purpose, collective identity, distributed leadership, visibility, and ongoing participation [9,10]. These processes may be especially important to EBP projects that rely on voluntary engagement, peer pressure, and continuous commitment across professional groups.

The RNAO Best Practice Spotlight Organization^®^ (BPSO) initiative is a formal knowledge translation approach to implementation of best practice guidelines in healthcare and academic settings [11,12]. In the Philippines, a university-affiliated medical center and College of Nursing used this approach to support guideline implementation through training, capacity building, and integration of EBP across clinical and educational activities.

This paper describes a multi-year EBP implementation initiative within this Philippine academic–clinical partnership. Using the Social Movement Action Framework, we examined how preconditions for change, social movement mechanisms, and implementation outcomes were reflected in the initiative. The purpose was not to test effectiveness, but to describe how social movement strategies may help explain the engagement, shared ownership, and implementation momentum observed in this setting.

## 2. Conceptual Framework

This initiative is guided by the Social Movement Action Framework, which views large-scale change as emerging through shared purpose, collective action, and sustained participation [10]. This perspective aligns well with implementation science, as EBP implementation rarely depends solely on evidence. It also depends on whether people see the change as meaningful, whether they have support from their organization, and whether the change can be adapted to the setting where care is delivered [5,9].

The framework includes three main domains: preconditions, key characteristics, and outcomes. Preconditions are the conditions that make change possible. In this initiative, these included recognition of a practice gap, readiness for change, and alignment with the values of both the academic and clinical partners [13]. These conditions helped explain why faculty, students, and clinical staff were willing to engage in the work.

The key characteristics of a social movement include intrinsic motivation, emerging leadership, collective identity, visibility, and networks that provide support [9]. These characteristics are useful for understanding EBP implementation because change does not occur solely because a guideline is introduced or a policy is announced. People also need to believe that the work matters, see themselves as part of the change, and feel connected to others who are working toward the same goal.

Outcomes refer to what the movement can support over time, such as the adoption of new practices, integration into routine work, spread to other settings, and continued capacity for improvement. These outcomes are consistent with implementation science’s focus on sustainability and scale-up, rather than initial adoption alone [5].

In this manuscript, the Social Movement Action Framework is used to examine EBP implementation as a social process. It helps explain how the work moved beyond training and guideline dissemination and became a shared effort among faculty, students, clinical staff, and organizational leaders.

To clarify the contribution of the social movement lens, Table 1 compares the Social Movement Action Framework with three established implementation frameworks frequently used in healthcare. The comparison shows that social movement theory does not replace these frameworks. Instead, it adds a useful focus on how motivation, shared identity, peer influence, and distributed leadership help implementation efforts gain and maintain momentum. This is consistent with RNAO’s description of the Social Movement Action Framework as a complement to knowledge translation approaches that support knowledge uptake and sustainability.

**Figure 1:**
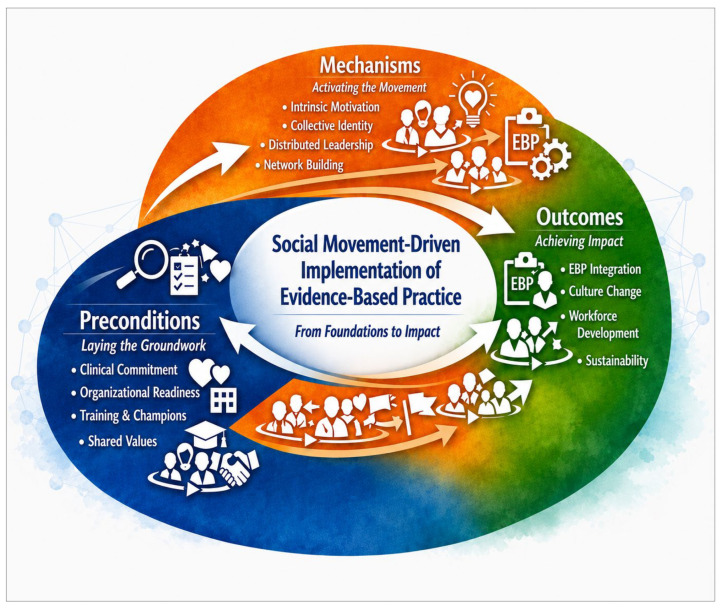
Social Movement-Driven Implementation of Evidence-Based Practice: From Preconditions to System-Level Outcomes.

Figure 1 presents the Social Movement-Driven Implementation of the EBP Model used in this case study. The model shows how readiness for change and alignment with institutional values can activate social movement mechanisms, including shared purpose, collective identity, distributed leadership, visibility, and network building. These mechanisms may then support implementation outcomes such as guideline adoption, capacity building, and sustained use of EBP in clinical and academic settings.

This figure presents a conceptual model of social movement-driven implementation, demonstrating how foundational preconditions activate key mechanisms that collectively drive the adoption, integration, and sustainability of evidence-based practice. The model highlights the dynamic interplay between individual motivation, collective identity, distributed leadership, and networked collaboration in fostering culture change, workforce capacity, and system-level transformation. Arrows indicate the dynamic, iterative relationships among domains, reflecting the non-linear nature of implementation processes.

## 3. Materials and Methods

### 3.1. Study Design

This paper used a descriptive case study approach to describe how EBP was implemented within an academic–clinical partnership in the Philippines. A case study approach was selected because the initiative occurred in a real-world setting and involved multiple interconnected activities, people, and organizations. Case study methods are useful when the goal is to understand a current practice or program within its actual context, especially when the boundaries between the program and the setting are closely connected [14].

The case was a multi-year EBP implementation initiative carried out by a university-affiliated medical center and its partner, the College of Nursing. The unit of analysis was the academic–clinical partnership and its shared work to implement best practice guidelines through the Registered Nurses’ Association of Ontario Best Practice Spotlight Organization^®^ (RNAO-BPSO) framework. The purpose was not to test clinical effectiveness, but to describe the implementation process and examine how social movement strategies were reflected in the work.

### 3.2. Setting and Case Description

The initiative was conducted in a university-affiliated hospital and a partner College of Nursing in the Philippines. These two institutions worked together to introduce and sustain best-practice guidelines across both clinical and educational settings. The work included champion training, guideline-related activities in hospital units, integration of EBP concepts into nursing education, and student participation in clinical and community-based activities.

The case was bounded by the academic–clinical partnership, the RNAO-BPSO implementation activities, and the period during which the initiative was planned, implemented, and sustained. The case was selected because it provided an example of EBP implementation in a low- and middle-income country context where academic and clinical partners worked together to support practice change.

### 3.3. Data Sources

Data came from existing records and materials related to the initiative. These included program and institutional documents, BPSO champion training materials and records, implementation activity reports, curriculum materials, student engagement records, and internal reports describing guideline adoption and practice integration. These materials were selected because they documented the activities of the academic and clinical partners.

The records were reviewed to identify what was done, who was involved, and how the implementation activities reflected the Social Movement Action Framework. The documents also helped identify examples of preconditions for change, social movement mechanisms, and implementation outcomes. Using several types of records helped provide a broader view of the initiative and supported triangulation across sources [15].

### 3.4. Data Review and Analysis

The data were reviewed using a framework-guided approach. The Social Movement Action Framework was used to organize the information into three main areas: preconditions for change, key social movement mechanisms, and implementation outcomes. This approach was chosen because the study aimed to understand how social movement strategies were reflected in the implementation process.

The authors reviewed available records and identified examples that fit each framework area. For example, information about readiness, leadership support, and alignment with institutional priorities was grouped under preconditions for change. Information about shared purpose, champion involvement, peer influence, visibility, and collaboration was grouped under social movement mechanisms. Information about guideline integration, continued training, and spread of EBP activities was grouped under implementation outcomes.

This was not a formal qualitative interview study. Therefore, procedures such as interview coding, member checking, or analysis of participant narratives were not used. Instead, the analysis focused on reviewing existing program materials and implementation records. Findings were checked across different sources when possible to improve credibility and reduce reliance on a single type of evidence.

### 3.5. Trustworthiness and Credibility

Several steps were taken to strengthen the case description’s credibility. First, the authors used multiple data sources, including training records, implementation reports, curriculum materials, and internal documents. Second, the findings were organized using an established framework, which helped keep the analysis focused and consistent. Third, the authors reviewed the findings in relation to both clinical and academic activities to avoid presenting the initiative from only one perspective.

Because the initiative was described using existing records, the study did not include interviews, focus groups, or direct collection of staff or student perspectives. This limited the ability to conduct member checking or formal reflexivity procedures. This limitation is acknowledged in the Discussion.

### 3.6. Ethical Considerations

This manuscript describes a practice improvement initiative that used existing program materials and implementation records. No identifiable patient, staff, or student data were collected or analyzed. Staff perspectives were not analyzed through interviews, focus groups, or survey data. Because the work describes a quality and practice improvement initiative using existing records, a formal institutional ethics review was not required. The manuscript and figures were polished and enhanced using generative AI tools to ensure a high-quality presentation.

### 3.7. Reporting Considerations

This manuscript was prepared as a descriptive implementation case study. Because the study did not involve interviews or focus groups, the Consolidated Criteria for Reporting Qualitative Research checklist was not fully applied [16]. Instead, the manuscript was guided by reporting principles relevant to implementation studies, including a clear description of context, implementation activities, data sources, and observed outcomes [17]. The goal was to provide enough detail for readers to understand the setting, the implementation process, and the limits of the available evidence.

## 4. Results

The findings are organized according to the Social Movement Action Framework as depicted in Figure 1: preconditions for change, key social movement mechanisms, and implementation outcomes. Because this was a descriptive case study, the results focused on observed implementation activities and process indicators rather than formal clinical outcome measures.

### 4.1. Preconditions for Change

Several conditions helped prepare the academic and clinical partners for EBP implementation. First, both groups recognized the need to strengthen the use of evidence-based guidelines in clinical practice and nursing education. This shared recognition created a common starting point for the initiative.

Leadership support was also important. Leaders from the university, College of Nursing, and partner medical center supported participation in the RNAO-BPSO program. They encouraged faculty, clinical staff, and students to take part in training and implementation activities. Early activities included BPSO champion training, review of practice gaps, and planning for guideline-related work in selected clinical and educational areas.

The initiative was also aligned with the mission of both institutions. For the College of Nursing, the work supported the preparation of students who could use evidence in clinical practice. For the medical center, it supported efforts to improve care processes and strengthen nursing practice. This alignment helped make the initiative relevant to faculty, students, and clinical staff.

Taken together, these preconditions suggest that the initiative began in a setting with readiness for change, leadership support, and shared interest in improving EBP.

### 4.2. Key Characteristics of the Social Movement

#### 4.2.1. Shared Purpose and Motivation

A shared purpose developed around improving patient care through EBP. Participants were not only responding to institutional expectations; they also appeared to see the work as meaningful to their professional roles. Faculty, students, and clinical staff did more than attend training or respond to formal expectations. They also took part in activities that created shared ownership, peer influence, and wider visibility for EBP.

#### 4.2.2. Collective Identity

The initiative helped create a shared EBP language across academic and clinical settings. Faculty, students, and clinical staff began using similar terms and concepts related to best-practice guidelines, gap assessment, implementation, and evaluation. This common language helped connect classroom learning with clinical practice. The development of a shared identity was also reflected in the role of BPSO champions. Champions were not limited to one group. Faculty, clinical nurses, and students all participated in activities that supported EBP. This helped frame the work as a shared responsibility rather than the work of one department or one group of leaders.

#### 4.2.3. Distributed Leadership

Leadership was not limited to formal administrators. Faculty members, staff nurses, and student champions each played a role in advancing the work. Faculty supported the integration of guidelines into learning activities. Clinical nurses helped apply guideline recommendations in practice areas. Students used guideline-informed approaches during clinical and community-based experiences.

This pattern reflects distributed leadership, in which implementation work was carried out by several groups rather than being directed solely from the top.

#### 4.2.4. Network Building and Collaboration

The academic–clinical partnership created a structure for collaboration. The College of Nursing and the medical center worked together through training, implementation planning, and guideline-related activities. These networks allowed faculty, students, clinical nurses, and leaders to share information and coordinate activities. The partnership also supported the movement of knowledge across settings. Students learned about EBP in academic settings and then used guideline-informed approaches during clinical and community experiences. Clinical staff and faculty also worked together to support guideline implementation in practice areas.

#### 4.2.5. Visibility and Momentum

The initiative became more visible through training sessions, workshops, champion activities, and guideline-related projects. These activities helped introduce the work to more people and created opportunities for wider participation. Visibility also helped maintain momentum. As more faculty, students, and clinical staff became familiar with the initiative, the work became easier to recognize as part of the organization’s EBP efforts. This visibility supported continued engagement, although the study did not formally measure changes in motivation or participation over time.

#### 4.2.6. Individual and Collective Action

The initiative included both individual and group actions. Individual participants attended training, served as champions, and applied guideline-informed practices in their own areas. At the group level, academic and clinical partners worked together to identify practice gaps, plan implementation activities, and support guideline use. These actions show how implementation moved beyond awareness of EBP and into practice-related activities across academic and clinical settings.

### 4.3. Implementation Outcomes

#### 4.3.1. Adoption and Integration of EBP

The initiative supported the adoption of EBP through champion training, guideline-related activities, and the integration of best-practice concepts into nursing education and clinical practice. In the academic setting, EBP was incorporated into selected learning activities, clinical preparation, and student engagement activities. In the clinical setting, guideline-related work focused on areas such as breastfeeding support, vascular access device management, and pressure injury prevention. These activities provide process indicators of EBP adoption and integration. However, the study did not include formal measurement of adoption rates or clinical outcomes.

#### 4.3.2. Development of an EBP-Oriented Culture

The initiative appeared to support a stronger EBP orientation among participants. This was reflected in the use of common EBP language, continued champion involvement, and the inclusion of guideline-related activities in both academic and clinical settings. Because the study did not include surveys or interviews, the term “culture change” is used cautiously. In this case, culture change refers to observable signs that EBP became more visible and more regularly discussed within the academic–clinical partnership.

#### 4.3.3. Capacity Building and Workforce Development

The initiative contributed to capacity building by training faculty, clinical nurses, and students as champions. These champions supported EBP activities in classrooms, clinical areas, and community-based settings. Student involvement was especially important because it connected workforce preparation with clinical practice. These activities suggest that the partnership helped prepare future nurses to understand and participate in EBP implementation. The study did not formally measure long-term workforce outcomes, but the initiative created structures that supported EBP learning and practice.

#### 4.3.4. Sustainability and Spread

Evidence of sustainability was reflected in continued training activities, ongoing champion involvement, and the continued use of guideline-informed activities across academic and clinical settings. The initiative also showed signs of spread as EBP-related work was applied in more than one practice area and involved different groups of participants. These findings should be interpreted as indicators of sustainability and spread. The study did not include long-term outcome tracking or formal sustainability measures.

#### 4.3.5. System-Level Alignment

The initiative helped strengthen alignment between nursing education and clinical practice. Faculty, students, clinical nurses, and leaders participated in related activities that connected classroom learning with bedside and community-based care. This alignment supported a more coordinated approach to EBP implementation. The findings do not establish system-level transformation or clinical effectiveness. Instead, they show how an academic–clinical partnership created structures and activities that supported EBP implementation over time.

Overall, the results suggest that social movement strategies were reflected in the initiative’s development of shared purpose, distributed leadership, visibility, collaboration, and continued participation. These findings provide descriptive evidence of implementation processes, but they should not be interpreted as proof of clinical effectiveness.

## 5. Case Illustration: Implementation of Evidence-Based Guidelines in Clinical Practice

This case example shows how the social movement mechanisms described above were reflected in the implementation of selected RNAO Best Practice Guidelines within the partner medical center and College of Nursing. The example is included to show how the initiative moved from training and planning into practice-related activities.

### 5.1. Preconditions for Change

The initiative began when clinical and academic partners recognized the need to strengthen the use of evidence-based guidelines in practice. The medical center and College of Nursing formally joined the RNAO-BPSO program, which provided a structure for training, guideline implementation, and evaluation.

The first BPSO Champions training was held from August 29 to 1 September 2018. Faculty members from the College of Nursing and nurses from several hospital departments participated. The training introduced key steps in implementation, including gap analysis, readiness assessment, implementation planning, and evaluation. Support from academic and clinical leaders helped align the work with institutional goals and encouraged early participation from faculty, staff nurses, and students.

### 5.2. Social Movement Mechanisms in Action

After the initial training, implementation activities focused on selected clinical priorities, including vascular access device management, pressure injury prevention, and breastfeeding support. These areas were chosen because they were relevant to patient care and could be addressed through guideline-informed practice changes.

Implementation was carried out by champions, staff nurses, faculty, and students. Champions helped identify practice gaps, adapt guideline recommendations to local settings, and encourage their peers to use EBPs. This reflected distributed leadership, as responsibility for implementation was shared across groups rather than assigned solely to formal leaders.

The initiative also created opportunities for collaboration between the College of Nursing and the medical center. Faculty helped connect guideline content with teaching and clinical learning activities. Students applied guideline-informed practices during clinical and community experiences. Clinical staff supported the use of guidelines in practice areas. These activities helped move EBP across academic and clinical settings.

The breastfeeding guideline provides one example of how the work became visible. Champions supported breastfeeding-related activities, including advocacy for breastfeeding stations, breastmilk expression breaks, and milk banking. Breastfeeding education was also included in student learning activities, covering proper latch and positioning. These activities addressed observed gaps, such as inconsistent breastfeeding information and limited prenatal breastfeeding support.

### 5.3. Implementation Outcomes

The case demonstrated several implementation indicators. These included continued champion involvement, the use of guideline-informed activities in selected clinical areas, the integration of EBP content into student learning, and ongoing collaboration among faculty, students, and clinical staff. These activities suggest that the initiative helped strengthen the connection between nursing education and clinical practice.

The available records also showed that EBP-related activities were not limited to one group. Faculty, staff nurses, and students each played a role in supporting guideline implementation. Student champions reinforced EBP during clinical and community activities, while faculty and clinical partners connected guideline recommendations to practice expectations.

The case does not provide formal evidence of clinical effectiveness or long-term patient outcomes. However, it provides a concrete example of how social movement strategies were reflected in implementation activities. Shared ownership, champion involvement, peer support, and academic–clinical collaboration helped move the initiative from isolated training activities toward more regular use of guideline-informed practices.

Table 2 summarizes the implementation phases, key actions, actors involved, observable indicators, and social movement mechanisms reflected in this case example.

## 6. Discussion

This descriptive case study examined how social movement strategies were reflected in a multi-year EBP implementation initiative in a Philippine academic–clinical partnership. The findings suggest that shared purpose, champion involvement, distributed leadership, visibility, and collaboration helped support implementation activities across both clinical and educational settings. These findings should be interpreted as process-level evidence rather than evidence of clinical effectiveness.

The findings are consistent with recent implementation science literature, which shows that implementation is influenced by context, leadership, stakeholder engagement, and sustainability planning. For example, the updated Consolidated Framework for Implementation Research emphasizes that contextual factors can strongly affect whether implementation succeeds or fails in real-world settings [6]. Similarly, research has shown that implementation frameworks can help identify the core components and sustainability features of quality improvement work in healthcare settings [5]. Our case supports this view because implementation was not carried out solely through guideline dissemination. It depended on leadership support, academic–clinical alignment, champion training, and continued participation.

The findings also extend prior work by showing how social movement mechanisms may help explain the “people side” of implementation. Established implementation frameworks help identify barriers, facilitators, and implementation conditions. However, they may give less attention to how people become motivated to take part in change and continue the work over time. Grinspun et al. (2022) described social movement action for knowledge uptake and sustainability as voluntary, intrinsically motivated, and centered on a common cause [9]. Our case reflects this idea. Faculty, students, and clinical staff did more than attend training or respond to formal expectations. They also took part in activities that created shared ownership, peer influence, and wider visibility for EBP.

The case also aligns with studies of the RNAO-BPSO program in other settings. One study showed that leadership support, organizational culture, and local champions were important in establishing and sustaining a culture of EBP in the Australian healthcare context [12]. Rivas-González and colleagues similarly identified facilitators and barriers to implementing Best Practice Guidelines under the BPSO model, including the importance of organizational support, professional engagement, and implementation structures [11]. Our case confirms these findings in a Philippine academic–clinical setting. At the same time, it extends them by showing how academic partners, students, and clinical staff can participate together in guideline implementation.

This study also adds to the discussion on EBP implementation in resource-constrained or complex settings. Recent work has emphasized that implementation strategies must be adapted to local context and supported by practical structures that help people use evidence in everyday care [18,19]. In this case, the academic–clinical partnership helped connect EBP training with clinical and community-based activities. This connection may be important in settings where workforce development and practice improvement need to occur together.

Several alternative explanations should also be considered. The implementation activities described in this case may not have resulted only from social movement strategies. The structure of the RNAO-BPSO program may also have influenced them, including the commitment of institutional leaders, existing relationships between the College of Nursing and the medical center, and participants’ professional values. For this reason, the social movement lens should be viewed as one way to interpret the case, not as the only explanation for the observed implementation activities.

The initiative has important limitations. First, it is based on one academic–clinical partnership, so the findings may not apply to all healthcare settings. Second, the study used existing program records and implementation materials. It did not include interviews, focus groups, surveys, or a formal collection of participant perspectives. Third, the study did not measure clinical outcomes, adoption rates, fidelity, or long-term sustainability using standardized implementation outcome measures. Because of these limits, the findings cannot be used to claim that social movement strategies caused improved clinical outcomes. Instead, the study provides a descriptive account of how social movement mechanisms were reflected in EBP implementation.

Despite these limitations, the case offers a useful example of how EBP implementation can move beyond training and guideline dissemination. The findings suggest that implementation efforts may be strengthened when people feel connected to a shared purpose, when champions are supported, and when academic and clinical partners work together. Future studies should examine similar initiatives using stronger designs, including mixed methods, implementation outcome measures, and longer-term follow-up.

## 7. Conclusions and Future Directions

### 7.1. Conclusions

This descriptive case study showed how social movement strategies were reflected in an EBP implementation initiative within a Philippine academic–clinical partnership. The initiative included champion training, guideline-related activities, curriculum integration, and collaboration between faculty, students, clinical staff, and organizational leaders.

The findings suggest that shared purpose, distributed leadership, peer support, and visibility may help EBP implementation gain momentum in real-world healthcare settings. These strategies did not replace formal implementation structures, such as the RNAO-BPSO program. Rather, they appeared to support the “people side” of implementation by helping participants see the work as meaningful and shared.

Because the study did not include formal clinical outcome measures, the findings should be interpreted with caution. The case provides descriptive evidence of implementation processes, not evidence that social movement strategies caused improved clinical outcomes. Even so, the case may be useful for healthcare and academic organizations seeking practical ways to support EBP adoption and continued engagement.

### 7.2. Future Directions

Future studies should examine social movement-informed implementation strategies in other healthcare and academic settings. Studies with stronger designs, including mixed-methods designs and longer follow-up, may help determine how these strategies relate to implementation outcomes such as adoption, fidelity, reach, and sustainability.

Future research should also include more direct measures of practice change and patient care outcomes when possible. This may include tracking adherence to guidelines, training participation, unit-level adoption, clinical process indicators, and patient outcomes before and after implementation. These data would help clarify whether social movement strategies contribute to measurable improvements in care.

Finally, more work is needed to understand how academic–clinical partnerships can support EBP implementation. Nursing education programs may be important partners in this work because they help prepare future clinicians while also supporting practice improvement in clinical and community settings.

## Figures and Tables

**Table 1:** Comparison of implementation frameworks and the added contribution of a social movement lens.

Framework	Main Focus	Common Use in Implementation Studies	Added Value of the Social Movement Lens
**Consolidated Framework for Implementation Research**	Identifies contextual determinants that may influence implementation, including inner and outer settings, individuals, intervention characteristics, and the process [6].	Helps identify barriers and facilitators before, during, and after implementation.	Focuses on how shared identity, peer influence, and grassroots participation help stakeholders stay engaged.
**Promoting Action on Research Implementation in Health Services**	Emphasizes the relationship among evidence, context, and facilitation in successful implementation [7].	Helps guide facilitation strategies and assess whether the environment is ready for change.	Adds attention to how collective purpose and distributed leadership can strengthen facilitation beyond formal roles.
**Medical Research Council framework for complex interventions**	Guides the development, adaptation, evaluation, and implementation of complex interventions [8].	Helps researchers understand intervention complexity and evaluate implementation in context.	Adds attention to how implementation momentum develops through visibility, networks, and collective action.
**Social Movement Action Framework**	Focuses on preconditions for change, shared purpose, collective identity, distributed leadership, visibility, and sustained action [9].	Helps explain how people become engaged and how implementation efforts spread through social processes.	Provides a relational and motivational explanation for how EBP implementation may be sustained in real-world settings.

**Table 2:** Implementation phases, key actions, actors involved, observable indicators, and social movement mechanisms aligned with the conceptual model (Figure 1).

Phase	Key Actions	Actors Involved	Observable Indicators	Social Movement Mechanisms
**Preconditions for change**	Assessed readiness for EBP implementation; aligned the initiative with institutional priorities; identified early champions	Hospital leadership, nursing administrators, academic leaders, and faculty partners	Formal participation in the RNAO-BPSO program; initial BPSO Champions training; recognition of practice gaps; leadership support for implementation activities	Framing of shared purpose; early coalition building; legitimacy building
**Stakeholder mobilization**	Conducted awareness activities; introduced guideline-related priorities; recruited champions across academic and clinical settings	Clinical leaders, nurse managers, faculty educators, frontline nurses, and student participants	Participation of faculty and clinical staff in champion training; involvement of staff nurses and students in guideline-related activities; growing awareness of EBP priorities	Collective identity formation; peer influence; distributed leadership
**Collective engagement**	Organized training workshops; supported working groups; encouraged discussion of best practice guidelines in academic and clinical settings	Nurse champions, faculty members, students, clinical staff, and quality improvement teams	Champion-led activities; guideline discussions in learning and practice settings; shared EBP language across academic and clinical groups	Intrinsic motivation, network activation, and collaborative learning
**Practice integration**	Adapted guideline recommendations to local settings; connected guideline content with teaching, clinical learning, and selected practice areas	Clinical teams, faculty, students, nurse champions, and implementation partners	Use of guideline-informed activities in breastfeeding support, vascular access device management, and pressure injury prevention; integration of EBP content into student learning activities	Shared ownership; visible momentum; reinforcement of new practice expectations
**Sustainability mechanisms**	Continued champion involvement; maintained training and guideline-related activities; supported spread across academic and clinical settings	Hospital administrators, nurse champions, faculty educators, students, and professional development staff	Ongoing champion participation; continued use of guideline-informed activities; application of EBP in more than one practice area; continued academic–clinical collaboration	Institutionalization of practices; leadership continuity; diffusion of innovations

## Data Availability

No new data were generated or analyzed in this study.

## Abbreviations

The following abbreviations are used in this manuscript:

EBP	Evidence-Based Practice
RNAO	Registered Nurses’ Association of Ontario
BPSO	Best Practice Spotlight Organization
